# Sleep loss suppresses the motivation to prepare for sleep

**DOI:** 10.1101/2025.06.02.657276

**Published:** 2025-06-03

**Authors:** Nicole Freitag, Kareem El-Tawil, Aaron Crane, Vanessa Hilo, George Saad, Ada Eban-Rothschild

**Affiliations:** 1 Department of Psychology, University of Michigan, Ann Arbor, MI 48109, USA

**Keywords:** Pre-sleep behavior, nest-building, sleep deprivation, de-arousal, stress, machine learning

## Abstract

In the period preceding sleep, humans and other animals display a stereotyped repertoire of behaviors–including hygiene-related activities and preparing a place to sleep. Evidence suggests that this pre-sleep phase actively contributes to sleep initiation and quality. Nonetheless, individuals can sometimes fall asleep without preparation, even under undesirable circumstances. These abrupt transitions into sleep can have severe consequences, particularly in high-risk environments. Although progress has been made in identifying neuronal populations controlling sleep-wake states and mechanisms regulating cortical oscillations during sleep, little is known about the natural processes that govern the pre-sleep phase, under baseline conditions and following sleep loss. Here, we examine factors regulating pre-sleep behaviors using environmental and behavioral manipulations, video recordings, machine-learning-based tracking, and EEG-EMG analysis in freely-behaving mice. We focus on nest-building–a key pre-sleep behavior–and assess its modulation by time of day and sleep deprivation. We find that mice are highly motivated to build nests during the light phase but show reduced motivation during most of the dark phase. Sleep deprivation significantly suppresses pre-sleep nest-building and promotes the direct initiation of sleep. Varying amounts of sleep deprivation, from 2–6 hours, uniformly suppress nest-building. This suppression is not due to stress, as mice exposed to acute restraint stress engage robustly in nest-building. Our findings provide insight into processes regulating the transition from wakefulness to sleep. Understanding pre-sleep regulation has important implications for treating sleep-onset difficulties–prevalent in insomnia and predictive of cognitive decline–and for mitigating risks associated with uncontrolled sleep onset in high-stakes situations.

## INTRODUCTION

In the period immediately preceding the sleep phase, humans and other animals display a stereotypical repertoire of behaviors, including locating and preparing a safe sleeping area, performing hygiene-related behaviors, and assuming a sleeping posture^1–5^. The pre-sleep period is recognized as a transitional phase that promotes relaxation and actively contributes to the initiation and maintenance of sleep^6–12^. Nonetheless, individuals can sometimes fall asleep without any preparation, even under entirely undesirable circumstances, such as while driving. These abrupt transitions into sleep, typically attributed to sleepiness (e.g., ^13^), can have severe consequences for both the individuals involved and society when they occur in risk-prone environments. Although significant efforts have been made in recent decades to elucidate the brain mechanisms that regulate cortical oscillatory activity during sleep and pinpointing neuronal populations controlling sleep and wake states^7,14,15^, surprisingly little is known about the natural processes that govern the pre-sleep phase and the initiation of sleep under baseline conditions and following sleep loss.

Sleep timing, depth, and duration are understood to be regulated by two key processes: the circadian clock and the homeostatic sleep drive^16,17^. The circadian process generates a 24-hour cycle in physiology and behavior, setting the sleep/wake schedule^17–19^. In contrast, the homeostatic process is thought to gauge sleep need based on previous durations of wakefulness, with longer wake periods leading to increased sleep need^17,19,20^. While much effort has been directed toward elucidating the functions of circadian and homeostatic processes in the regulation of sleep timing, duration and depth, the impact of these processes on pre-sleep behaviors remains unexplored.

Our study aims to fill this knowledge gap by characterizing the factors that control behavioral preparation for sleep. To achieve this, we utilized environmental and behavioral manipulations, video recording, machine-learning-based tracking, and EEG-EMG analysis in freely behaving and sleeping mice. We determined the effect of time of day and sleep deprivation on the motivation to build a nest–a key pre-sleep behavior in mice^6,21^–locomotion, and sleep-wake architecture. We found that mice exhibit a strong motivation to build nests throughout the light phase, while showing little motivation at the beginning of the dark phase. Additionally, we found that sleep loss suppresses the motivation to build a nest prior to sleep, while also reducing locomotion and facilitating the direct initiation of sleep. Moreover, varying amounts of sleep deprivation–from 2 to 6 hours–uniformly suppress nest-building, suggesting that the mechanisms responsible for this behavioral suppression do not act proportionally to the amount of sleep lost, in contrast to the proportional nature of the homeostatic sleep rebound. Notably, stress alone does not account for this suppression of nesting behavior, as evidenced by the strong motivation to build a nest following acute restraint stress. Our findings significantly enhance current understanding of the processes regulating the transition from wakefulness to sleep. A better understanding of these phenomena is crucial, not only because abrupt and uncontrolled transitions into sleep can have severe consequences for both individuals and society, but also because difficulties in initiating sleep are prominent symptoms of insomnia^22^ and strong predictors of cognitive decline later in life^23,24^.

## METHODS

### Animals

We used in-house bred wild-type (WT) C57BL/6J mice (The Jackson Laboratory, Stock #: 000664) that were reproductively inexperienced and older than 8 weeks. The mice were maintained under a 12-hr light/dark cycle at 22 ± 1°C, and provided with compressed cotton ‘Nestlet’ material (Ancare, Bellmore, NY, U.S.A.) and ad libitum access to water and food. Throughout the experiments, mice were individually housed in custom Plexiglas recording chambers (28.6 (W) × 39.4 (L) × 19.3 (H) cm) mounted with USB cameras (1080p USB webcam, Angetube). All experimental procedures adhered to the US National Institutes of Health Guide for the Care and Use of Laboratory Animals and were approved by the University of Michigan’s Institutional Animal Care and Use Committee.

### EEG-EMG implantation

Mice were anesthetized using a ketamine-xylazine mixture (100 and 10 mg kg^−1^, respectively) delivered via intraperitoneal injection (IP). Following this, they were administered lidocaine and carprofen (4 mg kg^−1^ and 5 mg kg^−1^, respectively). Subsequently, the mice were positioned in a stereotaxic frame (David Kopf Instruments, Tujunga, CA, U.S.A.) and maintained under anesthesia with approximately 1% isoflurane in O_2_. Mice were fitted with two miniature screw electrodes (F00CE125, J.I. Morris miniature fasteners, Inc.) at coordinates AP = 1.6 mm, ML = −1.6 mm and AP = −2.5 mm, ML = −2.8 mm and two EMG wire electrodes (A S633, Cooner Wire) previously soldered to a 4-pin connector (custom). EMG electrodes were inserted between the trapezius muscles of the mice. The implant was anchored securely to the skull using C&B Metabond (Parkell) and dental cement, and the skin incision was closed using surgical sutures. After the surgery, the mice were placed on a heating pad until they regained full mobility. They were then given 7–10 days for recovery, after which they were transferred to individual open-top recording chambers and habituated to flexible EEG-EMG cables for an additional 10 days before data collection began.

### Sleep deprivation through environmental enrichment

Mice were placed in an enclosure containing bedding, water, and a running wheel, but without nesting material or food. They were continuously monitored by an experimenter in an adjected room using the iSpy software (iSpyConnect.com) and through their EEG-EMG signals when available. Whenever a mouse showed signs of quiescence, novel objects, such as wooden cubes and plastic figures, were introduced or exchanged in the enclosure. Sleep deprivation lasted 2–6 hours.

### Sleep deprivation through gentle handling

An experimenter continuously observed the mice in their home environment along with their EEG-EMG signals when available. Whenever the mice began to exhibit signs of quiescence, the experimenter gently stroked them with a soft brush or lightly tapped their cage. Sleep deprivation lasted for 4 hours.

### Acute stress

Prior to the nesting manipulation at either ZT 23.5 or ZT 5.5, test mice underwent 30 minutes of immobilization stress using a well-ventilated perforated 50-ml centrifuge tube.

### Nest manipulations and scoring

Manipulations were performed on mice that had resided in their home cages for at least three days. On experimental days, we removed the nests from the home cages of test mice and introduced new nesting material, consisting of four Nestlet strips weighing a total of 5 gr. The new nesting material was placed away from the original nest location. Throughout the nest manipulation experiment, we recorded the mice using USB cameras operated with the iSpy software (iSpyConnect.com). Repeated nest manipulations involving the same mice were conducted at least four days apart.

Two hours after the introduction of the new nesting material, we visually inspected the nests and photographed the cages from the top and the side. We also weighed any unshredded Nestlet material. Nest quality was assessed using a six-point scale, which was modified from the scale presented in Deacon, 2006:

1. Less than 5% of the Nestlets are shredded. The Nestlets strips are typically dispersed across the home cage or left undisturbed in the original location where they were placed.
2. Between 5–15% of the Nestlets are shredded. The Nestlets may be dispersed or gathered.
3. Between 15–30% of the Nestlets are shredded. The material is gathered within a quarter of the cage floor area, forming a discernible but flat nest.
4. 30–60% of the Nestlets are shredded and gathered to form either a platform or a cup-like structure. If a cup nest formation is present, the shredded material may be as little as 20%.
5. 60–80% of the Nestlets are torn and gathered. The walls of the nest are taller than the height of a mouse curled up on its side but cover less than 50% of the nest’s circumference.
6. More than 80% of the Nestlets are shredded. The nest resembles a crater with walls higher than a curled-up mouse, covering over 50% of its circumference.

### Polysomnographic acquisition and analysis

EEG-EMG signals were derived from the surgically implanted electrodes, amplified (Model 3500, A-M systems) and digitized at a rate of 256 Hz (Vital Recorder, Kissei Comtec America). Subsequently, the signal was filtered (EEG, 0–25 Hz; EMG, 25–50 Hz) and spectrally analyzed via fast Fourier transformation using either the SleepSign for Animal software (Kissei Comtec America), a modified version of the open-access Accusleep software ^25^ or custom MATLAB script (MathWorks, Natick, MA, U.S.A.).

The data was first annotated semiautomatically into 4-s epochs as wake, non-rapid eye movement (NREM) sleep and rapid eye movement (REM) sleep. The scoring was then visually inspected and validated based on the EEG-EMG waveforms and their respective power spectra, with corrections made as necessary. All scoring was done by investigators blind to the experimental manipulation. We defined wakefulness by a desynchronized, low-amplitude EEG paired with tonic EMG activity exhibiting phasic bursts. NREM sleep was defined by a synchronized, high-amplitude, low-frequency (0.5–4 Hz) EEG and a markedly diminished EMG activity relative to wakefulness. REM sleep was defined by diminished low-frequency oscillations, a prominent theta rhythm (4–9 Hz), and an absence of EMG activity. To determine the latency to NREM sleep, we identified the initial NREM sleep episode exceeding 12 seconds. Similarly, for REM sleep latency, we identified the first REM sleep episode surpassing 8 seconds.

### Manual analysis of nest-building behavior

We annotated mice nest-building behavior second-by-second using the Behavioral Observation Research Interactive Software (BORIS)^26^ from video data. Nesting was defined for instances where the mouse was engaged in pulling, carrying, fraying, push-digging, sorting or fluffing of nesting material, as in ^27^.

### Automatic locomotion analysis

We used the Detect Any Mouse Model (DAMM)^28^ toolbox, which employs instance segmentation to detect and track the location of mice in video recordings, generating bounding boxes that define the position and size of each animal. The original DAMM model weights and configuration files were used in a zero-shot setting^28^. Using custom Python code, we calculated the centroid of each bounding box and computed the mean distance traveled in 10-minute bouts. To reduce noise from small posture-related shifts, a minimum displacement threshold of 0.5 cm per frame was applied, and only movements exceeding this threshold were included in the distance calculations.

### Figure and representative video preparation

Data plots were generated using either Prism 9.1 (GraphPad Software) or custom MATLAB (MathWorks, Natick, MA, U.S.A.) or Python scripts and were exported to Adobe Illustrator 2023 (Adobe Creative Cloud) for final figure preparation. Representative videos were created using Adobe Premiere Pro (Adobe Creative Cloud).

### Statistical analysis

**Figure 1.**
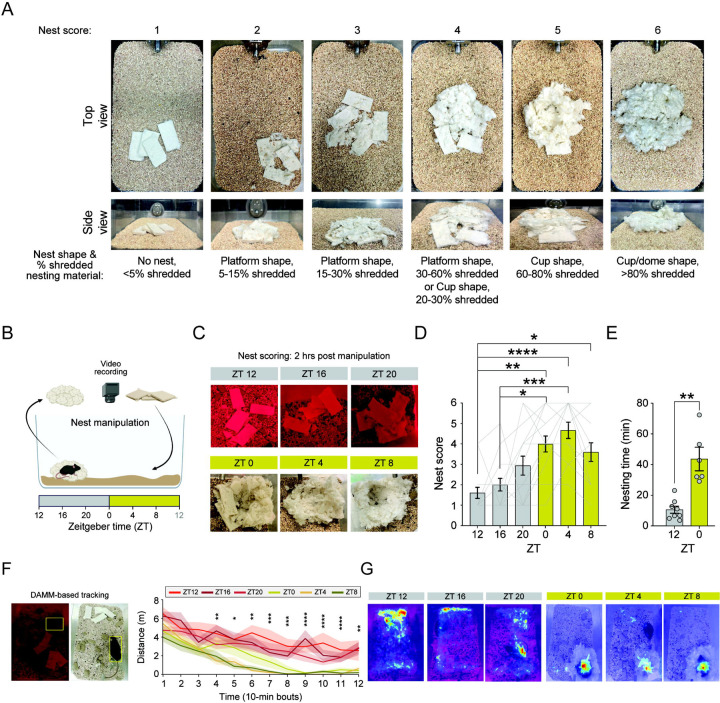
Mice show a strong motivation to build nests throughout the light phase. **(A**) Visual representation of nest scores, with home cage images from top and side views, along with the corresponding nest shape and percentage of nesting material used shown below each image. (**B**) Schematics of the nesting manipulation paradigm. Nests were removed from home cages at different circadian time points (Zeitgeber Time [ZT] 12, 16, 20, 0, 4 and 8), and new nesting material (5 g Nestlet) was placed inside each home cage, away from the previous nest location. Nests were scored two hours post manipulation. Representative images of nests (**C**) and nest scores (**D**) of mice whose nesting material was manipulated at ZT 12, 16, 20, 0, 4 and 8. n = 15 mice. Friedman test followed by Dunn’s multiple comparisons. (**E**) Total time spent nesting during the 2-hour period following nest manipulation at ZT 12 and ZT 0. n = 8 mice. One-tailed paired t-test. (**F**) Distance traveled during the 2-hour post-manipulation period, calculated from bounding box centroids. Left: example snapshots showing mice and detected bounding boxes during the dark and light phases. Right: data binned into 10-minute intervals. Friedman tests with FDR-corrected post hoc Wilcoxon signed-rank tests. n = 10 mice. (**G**) Representative heatmaps showing bounding box centroid locations across the 2-hour post-manipulation period, following nesting manipulations at ZT 12, 16, 20, 0, 4 and 8. Data depicts mean ± SE.

**Figure 2:**
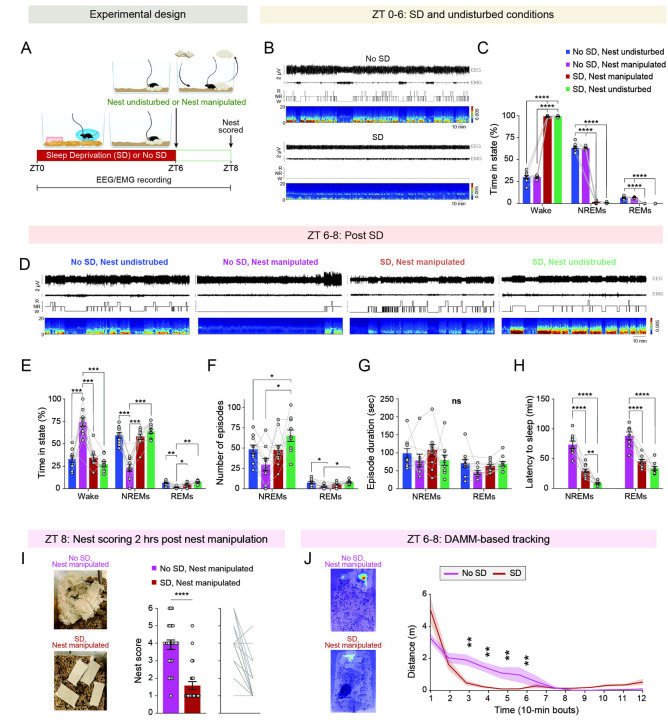
Six hours of sleep deprivation suppresses the motivation to build a nest prior to sleep. (**A**) Schematic of experimental design. Mice were subjected to six hours of sleep deprivation (SD) via environmental enrichment from zeitgeber time (ZT) 0–6, followed by nest manipulation at ZT 6. Control mice were either not subjected to SD prior to nest manipulation or were left with their original nests undisturbed. EEG, EMG and video data were recorded throughout, and nests were evaluated two hours after manipulation. (**B**) Representative 6-hour EEG and EMG traces, hypnogram, and spectrogram from a mouse subjected to SD (bottom) or left undisturbed (top). (**C**) Percentage of time spent in wake, NREM, and REM sleep during ZT 0–6 under sleep-deprived and undisturbed conditions. (**D**) Representative 2-hour EEG and EMG traces, hypnogram, and spectrogram from ZT 6–8 in mice subjected to different experimental conditions. (**E**) Percentage of time spent in wake, NREM, and REM sleep during ZT 6–8. (**F**) Number of wake, NREM, and REM sleep episodes during ZT 6–8. (**G**) Duration of wake, NREM, and REM sleep episodes during ZT 6–8. (**H**) Latency to NREM and REM sleep following the return of mice to their home cages after sleep deprivation and/or nest manipulation. Latency is not shown for mice that were neither sleep-deprived nor manipulated, as they were already asleep at ZT 6. (**I**) Nest scores 2 hours post nest manipulation (ZT 8) in mice that were either left undisturbed or subjected to 6 hours of SD during ZT 0–6. Left: representative nest images. Middle: nest scores. Right: individual data points. (**J**) Distance traveled during the 2-hour period following nest manipulation (ZT 6–8), calculated from bounding box centroids in mice that were either previously undisturbed or subjected to 6 hours of sleep deprivation. Left: heatmaps of centroid locations during the post-manipulation period. Right: distance data binned into 10-minute intervals. Data depicts mean ± SE. n = 10 mice. For data depicted in (C) and (E-H), we used RM mixed-effects ANOVA, followed when appropriate by Tukey’s multiple comparisons test. For data depicted in (I) and (J), we used a Wilcoxon matched-pairs signed-rank test. ns, p > 0.05; *, p < 0.05; **, p < 0.01; ***, p < 0.001; ****p < 0.0001.

**Figure 3.**
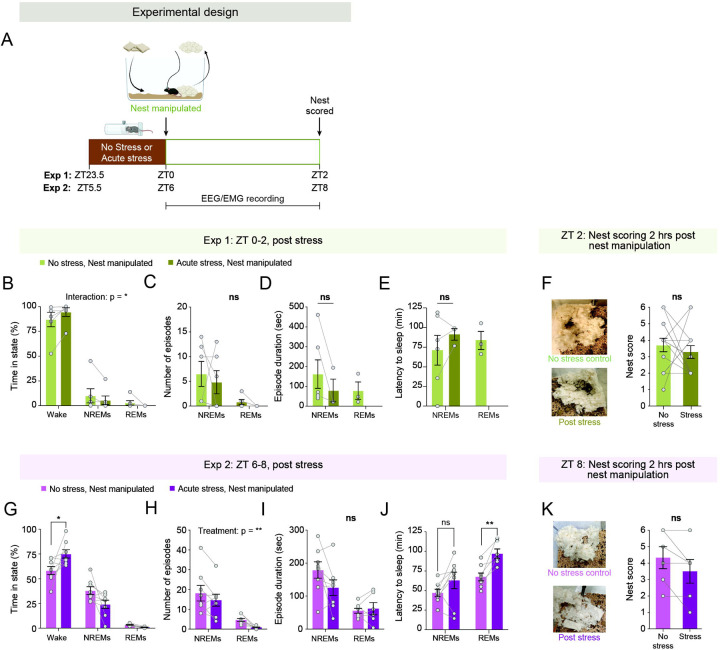
Acute stress does not suppress the motivation to build a nest. (**A**) Schematic of experimental design. Mice were subjected to 30 minutes of acute restraint stress prior to nest manipulation at either ZT 0 (n = 8 mice) or ZT 6 (n = 6 mice). Control mice remained undisturbed before nest manipulation. EEG, EMG, and video data were recorded throughout, and nests were evaluated two hours post nesting manipulation. (**B**) Percentage of time spent in wake, NREM, and REM sleep during ZT 0–2 in control and stress-exposed mice. (**F**) Number of wake, NREM, and REM sleep episodes during ZT 0–2 in control and stress-exposed mice. (**G**) Duration of wake, NREM, and REM sleep episodes during ZT 0–2 in control and stress-exposed mice. (**H**) Latency to NREM and REM sleep following nest manipulation at ZT 0. As some mice remained continuously awake following stress exposure or did not enter REM sleep, their data points for number of sleep episodes, episode duration, and latency to sleep are missing. (**F**) Nest scores 2 hours after nest manipulation (ZT 2). Left: representative nest images. Right: nest scores. (**G**) Same as (B), but during ZT 6–8. (**H**) Same as (C), but during ZT 6–8. (**I**) Same as (D), but during ZT 6–8. (**J**) Same as (E), but during ZT 6–8. (**K**) Same as (F), but at ZT 8. Two-way RM ANOVA followed by Šídák’s multiple comparisons test was used for (B–C) and (G–H). We used RM mixed-effects ANOVA, followed (when appropriate) by Tukey’s multiple comparisons test, for data depicted in (D–E) and (I–J). We used Wilcoxon matched-pairs signed-rank test for data depicted in (F) and (K). ns, p > 0.05; *p < 0.05; **p < 0.01; ***p < 0.001; ****p < 0.0001.

**Figure 4:**
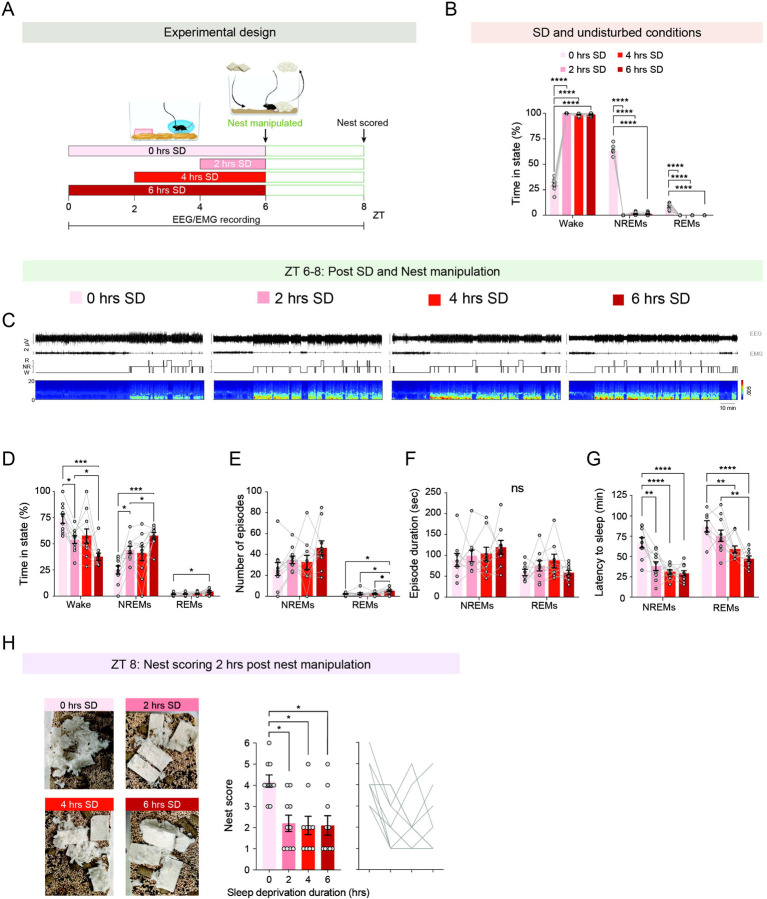
Varying durations of sleep deprivation uniformly suppress the motivation to build a nest prior to sleep. (**A**) Schematic of experimental design. Mice were subjected to 2, 4, or 6 hours of sleep deprivation (SD) via environmental enrichment, followed by nest manipulation at ZT 6. Control mice were not sleep-deprived prior to manipulation (‘0 hrs SD’). EEG and EMG were recorded throughout, and nests were evaluated two hours post nesting manipulation (ZT 8). (**B**) Percentage of time spent in wake, NREM, and REM sleep during the SD procedure. Data for the ‘0 hrs SD’ group correspond to the ZT 0–6 time window. (**C**) Representative 2-hour EEG and EMG traces, hypnogram, and spectrogram from ZT 6–8 in mice subjected to different experimental conditions. (**D**) Percentage of time spent in wake, NREM, and REM sleep during ZT 6–8 following 0, 2, 4, or 6 hours of SD. (**E**) Number of wake, NREM, and REM sleep episodes during ZT 6–8 following 0, 2, 4, or 6 hours of SD. (**F**) Duration of wake, NREM, and REM sleep episodes during ZT 6–8 following 0, 2, 4, or 6 hours of SD. (**G**) Latency to NREM and REM sleep following nest manipulation at ZT 6. (**H**) Nest scores 2 hours post nest manipulation (ZT 8) in mice subjected to 0, 2, 4, or 6 hours of SD. Left: representative nest images. Middle: nest scores. Right: individual data points. Data depicts mean ± SE. n = 10 mice. For data depicted in (B), (D), and (E), we used two-way RM ANOVA followed by Tukey’s multiple comparisons test. For the data in (F) and (G), we used RM mixed-effects ANOVA, followed in (G) by Tukey’s multiple comparisons test. For the data in (H), we used Kruskal–Wallis test followed by Dunn’s multiple comparisons test. ns, p > 0.05; *, p < 0.05; **, p < 0.01; ***, p < 0.001; ****p < 0.0001.

**Figure 5:**
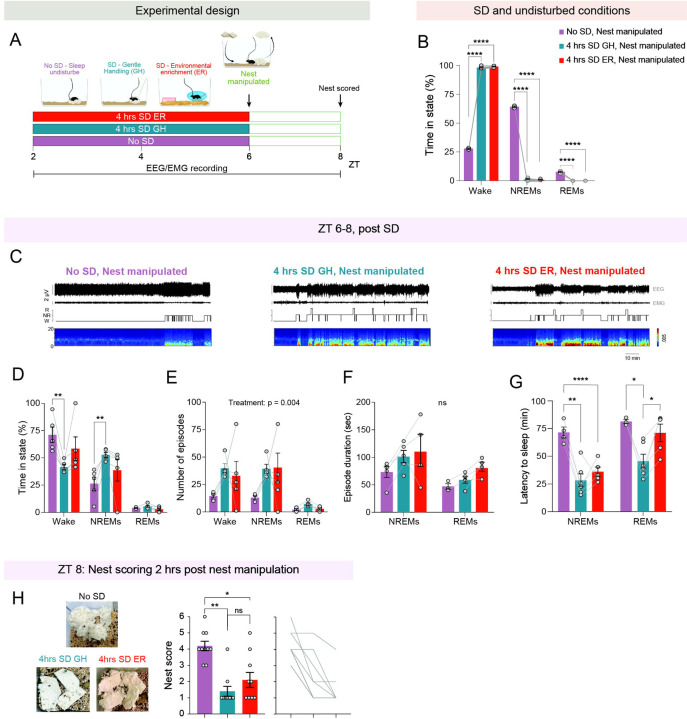
Sleep deprivation by gentle handling suppresses the motivation to build a nest similarly to sleep deprivation by environmental enrichment. (**A**) Schematic of experimental design. Mice were subjected to 4 hours of sleep deprivation (SD) during ZT 2–6 via either gentle handling or environmental enrichment, followed by nest manipulation at ZT 6. Control mice (‘No SD’) were left undisturbed prior to manipulation. EEG and EMG were recorded throughout, and nests were evaluated two hours post nesting manipulation (ZT 8). (**B**) Percentage of time spent in wake, NREM, and REM sleep during the SD procedure and undisturbed condition. (**C**) Representative 2-hour EEG and EMG traces, hypnogram, and spectrogram from ZT 6–8 in mice subjected to different experimental conditions. (**D**) Percentage of time spent in wake, NREM, and REM sleep during ZT 6–8. (**E**) Number of wake, NREM, and REM sleep episodes during ZT 6–8. (**F**) Duration of wake, NREM, and REM sleep episodes during ZT 6–8. (**G**) Latency to NREM and REM sleep following nest manipulation at ZT 6. (**H**) Nest scores 2 hours post nest manipulation (ZT 8). Left: representative nest images. Middle: nest scores. Right: individual data points. Data depicts mean ± SE. n = 6 mice for sleep architecture data, and n = 10 mice for nesting data. We used RM mixed-effects ANOVA, followed when appropriate by Tukey’s multiple comparisons test, for all data except (H). For the data shown in (H), we used the Friedman test followed by Dunn’s multiple comparisons. ns, p > 0.05; *, p < 0.05; **, p < 0.01; ***, p < 0.001; ****p < 0.0001.

Sample sizes were chosen based on prior publications investigating sleep/wake circuitry^6,29,30^. Non-parametric tests were applied for binned or scaled data and for datasets that did not meet normality assumptions. The Friedman test followed by Dunn’s multiple comparisons test was used for figures 1D and 5H. A one-tailed paired t-test was used for figure 1E. The Friedman test with FDR-corrected post hoc Wilcoxon signed-rank tests was used for figure 1F. Repeated-measures (RM) mixed-effects ANOVA, followed when appropriate by Tukey’s multiple comparisons test, was used for figures 2C, 2E–H, 3D–E, 3I–J and 4F–G. RM mixed-effects ANOVA, followed when appropriate by Šídák’s multiple comparisons test, was used for figures 5B, 5D–G. The Wilcoxon matched-pairs signed-rank test was used for figures 2I, 2J, 3F and 3K. Two-way RM ANOVA followed by Šídák’s multiple comparisons test was used for figures 3B–C and 3G–H. Two-tailed paired t-tests we used for figures 3D and 3E using. Two-way RM ANOVA followed by Tukey’s multiple comparisons test were used for figures 4B, 4D, and 4E. Kruskal–Wallis test followed by Dunn’s multiple comparisons test was used for figure 4H. We analyzed data using either Prism 9.1 (GraphPad Software) or custom MATLAB and python scripts.

## RESULTS

### Mice show a strong motivation to build a nest throughout the light phase

While the need for sleep varies throughout the day^16,17^, it remains unclear whether the motivation to prepare for sleep exhibits similar fluctuations. To explore this, we focused on nest-building behavior, a central pre-sleep activity in mice^6,21,30^. Nest-building can be readily quantified by assessing the shape of the nests and the weight of nesting material shredded within a specific time window (Fig. 1A and ^30,31^). Consequently, the quality of the nest can be used to infer the mice’s motivation to build it.

We subjected singly housed adult male and female mice to nest manipulation by replacing their old nests with new nesting material at various times across the light/dark cycle (Zeitgeber time (ZT) 12, 16, 20, 0, 4, and 8). This approach allowed us to evaluate the effects of both the phase of the light/dark cycle and time of day on their motivation to build a nest. We video-recorded the mice and assessed the quality of the nests two hours post-manipulation (Fig. 1B). We found that mice show a daily rhythm in their motivation to build a nest (Fig. 1C,D). During the dark phase, specifically at ZT 12 and 16, the mice show reduced motivation to build nests, minimally interacting with the provided nesting material (Fig. 1C,D). In contrast, mice demonstrate a consistently strong motivation to build nests across the light phase, with scores peaking at ZT 4 (Fig. 1C), despite the homeostatic drive for sleep being understood to decrease over this period as sleep need diminishes with time spent asleep^16,17^. To verify that our nest scoring scheme accurately represent the time spent nest building, we manually annotated the 2-hr long videos of mice following the nest manipulation conducted at ZT 12 and 0 (Fig. 1E). We found that mice spend significantly less time engaged in nest building at ZT 12 compared to ZT 0 (Fig. 1E), as reflected in our nest scoring (Fig. 1D). Together, these findings strongly suggest that mice exhibit a daily oscillation in their motivation to build nests, maintaining consistently high motivation throughout the entire light phase.

Next, using the machine-learning-based mouse tracking system DAMM^28^, we characterized locomotion in our video data during the 2-hour period following nest manipulations conducted at six different time points across the light/dark cycle (Fig. 1F). At all ZT times, nest manipulation was followed by high levels of locomotion lasting approximately 30 minutes (Fig. 1F and Table 1). However, beyond this time point, locomotion progressively decreased when manipulation occurred during the light phase, differing significantly from the dark-phase conditions (Fig. 1F and Table 1). Moreover, during the light phase, mice predominantly resided in the final nest location (Fig. 1G; summary data not shown). Together, these findings suggest that nest manipulation induces a transient increase in locomotion regardless of time of day, but during the light phase, this activity subsides once nest-building is completed and mice become quiescent inside their nests.

### The motivation to build nests prior to sleep is suppressed by six hours of sleep deprivation

Given that fatigue in humans is known to increase the likelihood of abrupt transitions from wakefulness to sleep (e.g., ^13^)–eliminating the behavioral preparation for sleep–we wondered whether sleep loss would similarly suppress the motivation to prepare for sleep in mice. To investigate this, we implanted adult female and male mice with EEG-EMG recording electrodes. After recovery, habituation, and single housing, mice were subjected to six hours of sleep deprivation via environmental enrichment, beginning at ZT0 and continuing through ZT6 (Fig. 2A–C). The procedure involved placing each mouse in an enclosure with bedding, water, and a running wheel—but without nesting material or food—and introducing novel objects (e.g., plastic figures) whenever the mouse showed signs of quiescence. We then (at ZT6) performed nest manipulations, as described above, and assessed the quality of the nests two hours post-manipulation (Fig. 2A, D–I). As controls, we subjected mice to three conditions: (1) six hours of undisturbed sleep followed by nest manipulation, (2) six hours of sleep deprivation followed by no nest manipulation, and (3) an entirely undisturbed condition: six hours of undisturbed sleep followed by no nest manipulation. EEG-EMG and video data were recorded throughout.

We first validated the effectiveness of our sleep deprivation procedure and found that mice subjected to sleep deprivation remained predominantly awake (98.94 ± 0.25% time awake) throughout the entire 6-hour procedure, spending very little time asleep compared to mice in the two undisturbed sleep conditions (Fig. 2B,C). Next, we characterized the sleep/wake architecture during ZT 6–8, the two hours following nest manipulation for the mice undergoing these manipulations (Fig. 2D–H). Mice subjected to nest manipulation after six hours of undisturbed sleep spent more time awake and less time in both NREM and REM sleep compared to the entirely undisturbed group (Fig. 2D,E). The increase in time spent awake was due to a decrease in the number of NREM sleep episodes, rather than an alteration in the mean duration of NREM sleep episodes (Fig. 2F,G). Notably, six hours of sleep deprivation suppressed these effects of nest manipulation on sleep/wake states, with the mice exhibiting decreased wakefulness and increased NREM and REM sleep, compared to mice subjected to nest manipulation following undisturbed sleep (Fig. 2F–G). Furthermore, the percentage of time spent in each state was similar between mice subjected to both sleep deprivation and nest manipulation and those not subjected to nest manipulation, regardless of whether they were undisturbed or sleep-deprived (Fig. 2E). Nonetheless, nest manipulation following sleep deprivation increased the latency to sleep compared to sleep deprivation alone and resulted in fewer episodes of NREM sleep (Fig. 2G,H). Together, these results suggest that sleep deprivation suppresses the arousal response induced by the need to prepare for sleep.

We next examined the effects of sleep deprivation on the motivation to build a nest. Notably, sleep-deprived mice had significantly lower nest scores two hours post-manipulation compared to mice that underwent nest manipulation at the same circadian time following undisturbed sleep (Fig. 2I). Moreover, mice subjected to nest manipulation after sleep deprivation exhibited a significant reduction in locomotion within 30 minutes of the manipulation, whereas non–sleep-deprived mice showed a marked decrease only after one hour (Fig. 2J). Together, these findings strongly suggest that sleep deprivation suppresses the motivation to build a nest prior to sleep initiation, reducing locomotion and directly promoting sleep.

### Acute stress does not account for the reduction in nest-building motivation following sleep deprivation

Since sleep deprivation not only suppresses sleep but also alters homeostasis and induces stress, we wondered whether the reduced motivation to build a nest following sleep deprivation might be solely a consequence of stress exposure rather than sleep loss. To test this hypothesis, we subjected singly housed female and male mice implanted with EEG-EMG recording electrodes to 30 minutes of acute restraint stress immediately prior to nest manipulation at ZT 0 (as described above) (Fig. 3A). EEG-EMG and video data were recorded throughout, and nest quality was assessed two hours post-manipulation.

We first examined sleep-wake architecture during the 2-hour period following nest manipulation. Overall, regardless of stress exposure, mice subjected to nest manipulation at ZT 0 were predominantly awake and spent little time in NREM and REM sleep during the test period (Fig. 3B–E). We next assessed the effects of acute restraint stress prior to nest manipulation on the motivation to build a nest. Notably, stress exposure did not alter this motivation, as stressed mice built nests of comparable quality to those of control mice and achieved similarly high scores (Fig. 3F). These findings suggest that stress, *per se*, does not suppress the motivation to build a nest.

We further wondered whether the time of day might influence the effect of acute stress on nest building, since our nest manipulations following sleep deprivation were conducted at ZT 6. We therefore repeated the experiment, exposing mice to acute restraint stress from ZT 5.5 to 6 and conducting nest manipulation at ZT 6 (Fig. 3A). During ZT 6–8, mice exposed to stress spent more time awake compared to non-stressed controls (Fig. 3G). Stress exposure led to a reduction in the number of both NREM and REM sleep episodes, without significantly affecting their mean duration (Fig. 3H–I). REM sleep latency was significantly increased following stress, while a non-significant trend was observed for NREM sleep (Fig. 3J). These findings suggest that acute stress promotes arousal following nest manipulation, but this effect may have been masked at ZT 0, when control mice were already predominantly awake after the manipulation.

We then examined the effects of stress at this time point on nest quality. Notably, as observed at ZT 0 (Fig. 3F), stress exposure did not affect the motivation to build a nest at ZT 6, as stressed mice built nests of comparable quality to those of control mice (Fig. 3K). Taken together, these findings strongly suggest that sleep loss—rather than acute stress—suppresses the motivation to build a nest prior to sleep.

### Even brief episodes of sleep deprivation, as little as two hours, suppress the motivation to build nests prior to sleep

We next wondered how different durations of sleep deprivation affect pre-sleep nest-building behavior. To address this, we subjected singly housed female and male mice implanted with EEG-EMG recording electrodes to nest manipulations immediately following 0, 2, 4, or 6 hours of sleep deprivation via environmental enrichment (Fig. 4A). All sleep deprivation procedures ended at ZT 6 to control for potential circadian effects on nest-building behavior. EEG-EMG and video data were recorded simultaneously.

We first validated the effectiveness of our sleep deprivation procedure and found that mice subjected to 2, 4, and 6 hours of sleep deprivation remained predominantly awake (99.2 ± 0.22% time awake) throughout the entire procedure, spending very little time asleep compared to mice in the undisturbed sleep condition (Fig. 4B). Next, we characterized the sleep/wake architecture during ZT 6–8, the two-hour period following nest manipulation (Fig. 4C–G). We found that the duration of sleep deprivation affected sleep/wake architecture: 2 hours of deprivation resulted in more sleep than 0 hours, but less than 6 hours (Fig. 4D). NREM sleep latency following 2, 4, and 6 hours of deprivation was similarly shorter than after 0 hours of sleep deprivation, while REM sleep latency was proportionally related to the duration of sleep deprivation (Fig. 4G). Next, we examined nest quality following the different durations of sleep deprivation. Surprisingly, we found a similar suppression of nest-building motivation following 2, 4, and 6 hours of sleep deprivation, with nest scores significantly reduced compared to those observed following undisturbed sleep (Fig. 4H). Together, these findings suggest that while sleep debt proportionally modulates sleep/wake architecture, its accumulation uniformly suppresses the motivation to prepare for sleep.

### Environmental enrichment does not account for the suppression of nest-building motivation following sleep deprivation

Lastly, we wondered whether the enriched experience during sleep deprivation, rather than sleep loss itself, could account for the suppression of nest-building motivation. To test this, we subjected singly housed female and male mice implanted with EEG-EMG recording electrodes to 4 hours of sleep deprivation via gentle handling, from ZT 2 to 6, followed by nest manipulation at ZT 6 (as described above) (Fig. 5A). The sleep deprivation via gentle handling was conducted in the home cage and did not involve the introduction of any salient stimuli, aside from the occasional brush used to gently arouse the mouse. As controls, mice were either allowed to sleep undisturbed or were sleep-deprived for the same duration using environmental enrichment. EEG-EMG and video data were recorded throughout, and nest quality was assessed two hours post-manipulation.

We first validated the effectiveness of our sleep deprivation procedures and found that mice subjected to 4 hours of sleep deprivation via either gentle handling or environmental enrichment, remained predominantly awake (98.74 ± 0.3% time awake) throughout the procedure, spending very little time asleep compared to mice in the undisturbed sleep condition (Fig. 5B). Next, we characterized sleep/wake architecture during ZT 6–8 (Fig. 5C–G) and found that wakefulness was reduced and NREM sleep was increased following sleep deprivation–particularly when conducted via gentle handling–compared to nest manipulation after undisturbed sleep (Fig. 5D). Sleep deprivation by either method similarly reduced the latency to NREM sleep following nest manipulation, relative to the undisturbed condition (Fig. 5G). However, the latency to REM sleep was shortened only after sleep deprivation via gentle handling, but not after deprivation by environmental enrichment (Fig. 5G). Finally, we examined nest quality following the different methods of sleep deprivation. Notably, we found that 4 hours of sleep deprivation suppressed the motivation to build a nest regardless of the deprivation method, with nest scores significantly reduced compared to those observed following undisturbed sleep (Fig. 4H). This strongly suggests that sleep loss–rather than the enriched experience during the deprivation procedure–is responsible for suppressing the motivation to build a nest prior to sleep.

## DISCUSSION

Our study reveals that the motivation to engage in pre-sleep nest-building behavior in mice follows a daily rhythm. Mice consistently show high nest-building motivation during the light phase–despite variations in homeostatic sleep drive–and markedly reduced motivation during the dark phase. By systematically excluding alternative explanations, we demonstrate that sleep loss suppresses the motivation to build a nest prior to sleep initiation, preventing the organism from organizing a safe sleeping environment and instead imposing sleep directly. Furthermore, we show that varying amounts of sleep loss uniformly suppress nest-building, suggesting that preparatory behaviors are actively regulated by mechanisms distinct from those governing the sleep process itself. Our work significantly advances current understanding of the processes that regulate the transition from wakefulness to sleep. A deeper understanding of the process of falling asleep is important—not only because abrupt and uncontrolled transitions into sleep can have serious consequences for individuals and society, but also because difficulty initiating sleep is a core symptom of insomnia^22^ and a strong predictor of cognitive decline later in life^23,24^.

By examining the motivation to build a nest at six time points across the 24-hour light/dark cycle, we show that mice exhibit a clear daily rhythm in their drive to engage in pre-sleep nest-building behavior. This rhythm appears to be more closely linked to the timing of sleep than to the homeostatic sleep need, as nest-building motivation remains high throughout the light phase—even as sleep pressure naturally dissipates^16,17^. Furthermore, our sleep deprivation experiments, which show a similar suppression of nest-building motivation following two, four, and six hours of deprivation, suggest that the sleep pressure induced by deprivation may differ qualitatively from that which accumulates during typical wakefulness in the active phase. How the neuronal substrates underlying homeostatic and circadian sleep drives interact with circuits mediating pre-sleep motivation remains a key open question.

While much effort over recent decades has focused on elucidating the roles of circadian and homeostatic processes in regulating sleep timing, duration, and depth, their influence on pre-sleep behaviors–and the neurobiological mechanisms that govern these behaviors–remains largely unexplored. This gap is striking, given that preparatory behaviors not only consistently precede sleep initiation but are also tightly and temporally aligned with it, suggesting that they are regulated by closely interacting physiological systems.

Several neuronal populations have been implicated in the regulation of pre-sleep nest-building behavior and sleep initiation, including dopaminergic neurons of the ventral tegmental area (VTA)^30^, somatostatin (SST)-expressing neurons in the medial prefrontal cortex (mPFC)^32^, and a glutamatergic subpopulation in the lateral hypothalamus (LH)^6^. VTA dopaminergic neurons regulate both sleep–wake states and behavioral preparation for sleep; reduced activity in these neurons permits pre-sleep behaviors such as nest-building, whereas their activation suppresses them^30^. In contrast, activation of SST-expressing mPFC neurons promotes both nest-building and sleep^32^. We have recently identified a glutamatergic neuronal subpopulation in the LH that regulates the motivation to engage in pre-sleep nest-building^6^. Chemogenetic activation of this ensemble promotes nest-building behavior, while its inhibition suppresses it^6^. Notably, suppressing the activity of these neurons also promotes sleep–but only when mice are confronted with the need to prepare a space in which to sleep^6^. In this context, activation of the ensemble promotes wakefulness, while inhibition leads to a rapid transition into sleep^6^. These findings suggest that this LH ensemble supports arousal and goal-directed, sleep-related behaviors specifically in the face of rising sleep pressure. Notably, our current findings on the suppression of nest-building following sleep loss closely resemble the effects observed with chemogenetic inhibition of this LH subpopulation^6^. Whether this behavioral suppression reflects a physiological inhibition of the same neuronal ensemble during sleep deprivation remains to be determined.

As sleep is a vulnerable state associated with reduced thermoregulation and increased exposure to predators and environmental threats, it is advantageous to restrict its occurrence to safe and ecologically appropriate settings. Preceding sleep with behaviors involving locating and preparing a sleeping area–that warrant that sleep is initiated under the proper conditions, such as within a nest–may represent an efficient evolutionary strategy. It is therefore striking that animals–including humans–can sometimes lose the ability to properly prepare for sleep, instead falling asleep abruptly even in dangerous situations. This phenomenon suggests that once sleep pressure rises beyond a critical level, the organism can no longer mount the appropriate arousal response needed to prepare for sleep or resist the drive to fall asleep, regardless of environmental context. Abrupt transitions into sleep likely reflect a fundamental physiological necessity; yet the neural mechanisms responsible for overriding sleep preparatory behaviors remain to be elucidated.

The *de-arousal model of sleep initiation*^7^ proposes that the expression of repetitive pre-sleep behaviors, such as grooming and nest-building, reduces vigilance toward the external environment, thereby lowering the tone of wake-promoting neuromodulators. This reduction in neuromodulatory tone leads to a brain-wide shift in excitability, functional connectivity, and information flow, ultimately imposing sleep upon the organism^7^. Our current findings suggest that sleep loss bypasses this usual preparatory step and triggers sleep directly. Future studies could critically test this hypothesis.

Here, we provide the first in-depth characterization of the relationship between sleep deprivation and pre-sleep behavior in mice, laying the groundwork for future mechanistic studies. Given that difficulty falling asleep and undesired transitions into sleep are widespread concerns in multiple sleep and neurodegenerative disorders^33^, as well as during aging–and are predictors of cognitive decline later in life^23,24^–a deeper understanding of the neural mechanisms that regulate the natural transition into sleep, and how these processes are altered under conditions of sleep loss, is of significant interest for the development of more effective therapeutic strategies.

## Data Availability

All code will be available via GitHub. All data will be provided upon reasonable request. Any additional information required to reanalyze the data reported in this paper is available from the lead contact upon request.

## STATEMENT OF SIGNIFICANCE

Prior to sleep, animals–including humans–engage in stereotyped behaviors such as preparing a sleep space, thought to facilitate sleep initiation and quality. However, sleep can occur abruptly without preparation, posing serious risks, especially in high-stakes environments. Despite progress in understanding sleep-wake circuitry, the regulation of pre-sleep behaviors remains poorly understood. Here, we used multiple techniques in mice to examine nest-building, a key pre-sleep behavior. We found that mice are more motivated to build nests during the light phase, but this behavior is strongly suppressed by sleep deprivation–regardless of duration–while not affected by acute stress. These findings highlight pre-sleep behaviors as an active, regulated component of sleep onset, with implications for understanding and treating sleep initiation difficulties.
